# Environmental influence on the choice of medicinal animals: a case study from northeastern Brazil

**DOI:** 10.1186/s13002-019-0337-9

**Published:** 2019-11-27

**Authors:** Iamara da Silva Policarpo Brito, Anna Karolina Martins Borges, Sérgio de Faria Lopes, Thelma Lúcia Pereira Dias, Rômulo Romeu Nóbrega Alves

**Affiliations:** 1 Programa de Pos-Graduacao em Ciências Biológicas (Zoologia), Departamento de Sistemática e Ecologia da Universidade Federal da Paraíba, João Pessoa, PB 58051-900 Brazil; 2 Departamento de Biologia, Universidade Estadual da Paraíba, Av. das Baraúnas, 351/Campus, Universitário, Bodocongó, Campina Grande, PB 58109-753 Brazil

**Keywords:** Folk medicine, Zootherapy, Medicinal animals

## Abstract

**Background:**

Animals from various taxonomic groups are commonly used in folk medicine, and their selection seems to be directly linked to their availability and accessibility. In the present study, we analyzed the use of animals as a source of folk medicines in a community in northeastern Brazil with access to aquatic and terrestrial animals. We hypothesize that the medicinal fauna is well represented by species of both habitat types.

**Methods:**

For the collection of information, semi-structured questionnaires were applied to local residents.

**Results:**

We recorded the use of 22 animals used as medicinal resources in the community, distributed among eight taxonomic categories, which are used to treat 38 types of diseases. Of the therapeutic animals, 14 species are terrestrial and 8 species can be considered aquatic occurring in marine or estuarine habitats.

**Conclusions:**

Our data confirm the tendency of human communities to use affordable medicinal animals in local ecosystems. We also found that medicinal use represents a strategy of optimizing the use of resources and is related to the economic, historical, social, cultural, and ecological contexts in which the community is inserted.

## Background

Medicinal plants and animals have been used in virtually all cultures as a source of medicine [1–5]. Due to the extensive use of plant materials [6–10], traditional medicine is associated with herbalism. However, recent research on animal species used in folk medicinal practices worldwide shows that products derived from medicinal animals are used directly in the elaboration of natural remedies that are widely sought in folk medicinal practices [11–16] and involve a large number of species. For example, at least 1500 animal species have some medicinal use in folk Chinese medicine [17] and in Latin America, at least 584 species have been reported to be used in folk medicinal practices [18]. Worldwide, at least 284 reptiles and 47 amphibians [19], 110 primates [20], 108 mammalian carnivores [21], 266 marine invertebrates [11], and hundreds of terrestrial invertebrates are used in folk remedies [22].

Although the use of animals for medicinal purposes is widespread and important in several aspects (e.g., cultural, economic, social, and ecological), the subject has been insufficiently researched when compared with medicinal plants [23]. Nevertheless, in the last 20 years, studies investigating the importance of animal use in folk medicine have become more frequent worldwide [12, 24–28] supporting the belief that animal use is widespread and present in the most diverse folk medical systems in the world [12].

In Brazil, a country with significant biological and cultural richness, many medicinal animals have been registered in several localities [29–31], especially in coastal communities [23, 32, 33] and in the semiarid region [34, 35]. These studies reveal that there is a tendency to select local species for use in folk medicine. Thus, people living in coastal areas tend to use mostly aquatic/marine resources while people from arid zones tend to use more animals and less aquatic resources [23, 33, 34, 36]. This situation is similar to that observed in the selection of plant species used by human communities, whose choice is influenced by their availability and accessibility [37].

Given the above, the aim of this article was to analyze the use of animals in medicinal practices of a fishing community in the district of Diogo Lopes, municipality of Macau, Rio Grande do Norte, Brazil. This fishing community is located in a peculiar region, where the dry forest extends to the coast. Therefore, the community has direct access to the animal resources that occur in a semiarid environment and the available fishing resources in the estuary and marine environment. Thus, the medicinal fauna used by the local population is expected to come from both the coastal area and the dry forest environments, since the diversity of environments enables the availability and access to a range of terrestrial and aquatic animals.

## Methods

### Study area

**Fig. 1:**
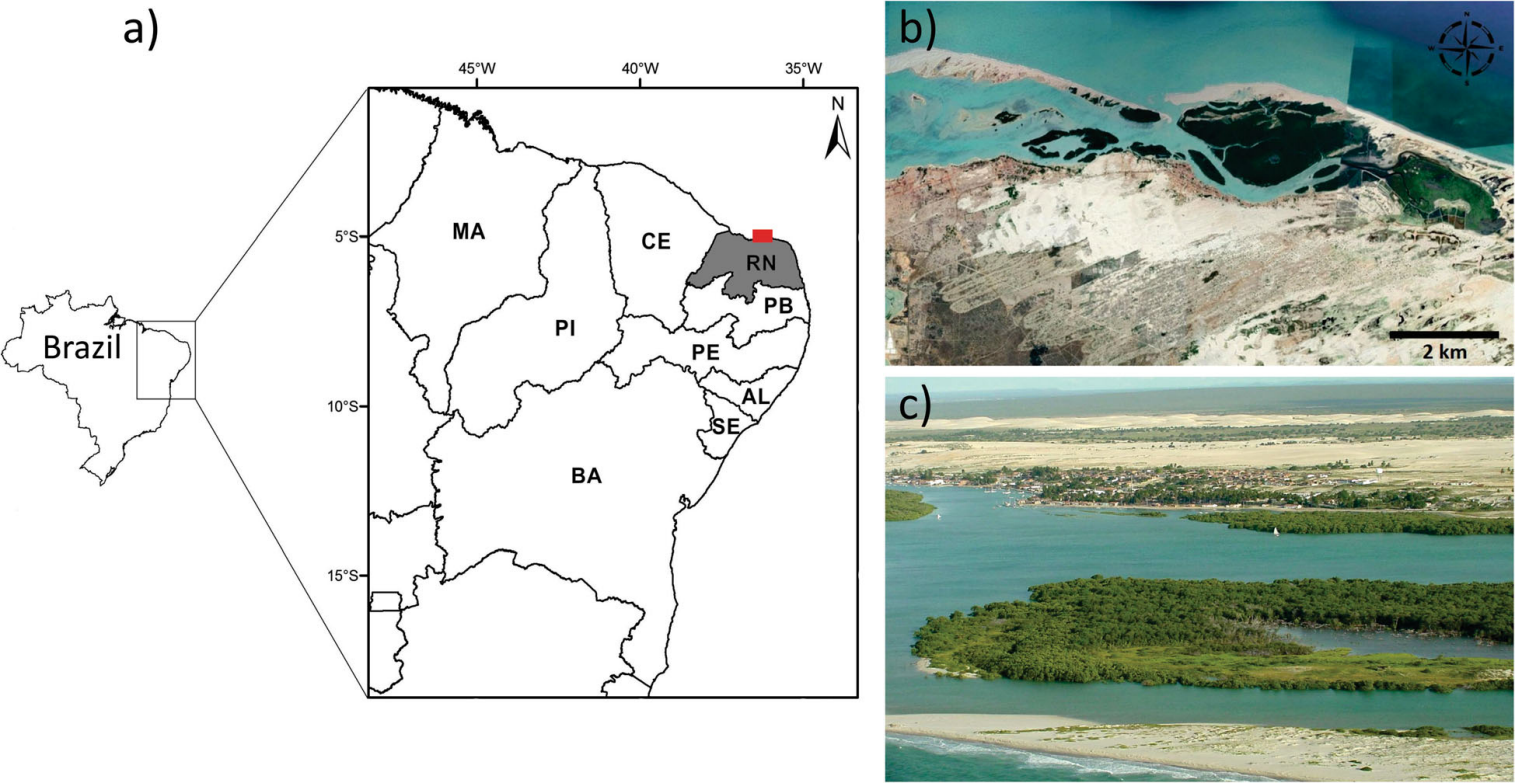
**a** Map of surveyed area, State of Rio Grande do Norte, northeast coast of Brazil. **b** Satellite image showing the coastal ecosystems and **c** partial view of the mangroves. Images: **b** Google Earth, **c** Getúlio Moura

Diogo Lopes district is part of the municipality of Macau, located approximately 185 km from the state capital of Rio Grande do Norte, Natal, Brazil (Fig. 1). The main access is through the road BR-406 and it covers an area of 100 km^2^ (Santos 2003). Data from the area is based on the Macao Meteorological Station, with latitude 5° 07′ S, longitude 36° 38′ W, and altitude of six meters [38]. According to ECOPLAM [39], the district of Diogo Lopes has a warm semiarid climate and terrestrial ecosystems are classified as dry forest, dune fields, and saline environments. The population is formed mostly by fishermen. Both the adult male and female populations live on the banks of the estuary where they benefit from fishing resources that are common to the region [40].

### Data collection

The execution of this research was reviewed and approved by the Research Ethics Committee of the Health Sciences Center of the Federal University of Paraíba (No. 2,244,394). Data collection was performed in the first semester of 2018. However, prior to the data collection, a presentation and clarification on the objectives of the research were given, and permission was granted by the interviewees to record the information obtained.

Information on the use of animals for medicinal purposes was obtained through semi-structured questionnaires, complemented by free interviews and informal conversations [41]. Additionally, during the interviews, the snowball technique was applied, which according to Baldin and Munhoz [42] is a non-probabilistic sample form used in social research in which the participants of a study indicate new participants who also indicate new participants and so on. Ninety-five people were interviewed, 50 females and 45 males. All individuals interviewed claimed to have used animals as a medicinal resource at some point in their lives.

The interviews were conducted individually, and the questionnaires contained questions about the name of the animal used for medicinal purposes, the parts used, diseases treated, ways of preparation and use, limitations of use, adverse effects, ways of obtaining the animal, efficacy of the medicine, preference between the zootherapic or synthetic, and indications of people who were also using zootherapy.

Vernacular names of species were recorded as cited by informants, and animals were identified as follows: (1) analysis of specimens donated by informants; (2) analysis of photographs of the animals (or their parts) taken during the interviews; (3) through the vernacular names, with the help of taxonomists familiar with the fauna of the study area. In the case of animals whose identification was not possible using the methods previously described, a specimen was collected for later identification.

The conservation status of these species was assessed through the Red List of Threatened Species of the International Union for the Conservation of Nature and Natural Resources (IUCN) [43] and the List of Brazilian Endangered Species [44].

### Data analysis

Initially, the data obtained from the interviews were organized into spreadsheets. In addition, all diseases treated by the abovementioned zootherapeutic drugs were grouped into 10 categories, based on the classification used by the Brazilian Center for Disease Classification (1993) as follows: (1) respiratory tract diseases (RTD); (2) digestive tract diseases; (3) undefined diseases; (4) external causes of morbidity and mortality; (5) diseases of the musculoskeletal system and connective tissue; (6) injuries, poisoning and some other consequences of external causes; (7) diseases of the circulatory system; (8) skin and subcutaneous tissue diseases; (9) nervous system diseases; and (10) ear and mastoid apophysis disorders (Table 1).

**Table 1:** Categories of diseases treated with zootherapeutic resources in the surveyed communities, according to CBCD - Brazilian Center for Classification of Diseases (1993)

Categories	Diseases	Total
Musculoskeletal and connective tissue disorders	Leg pain, plantar fasciitis, “hard nerves,” bone pain, joint pain, knee pain, hardened joints, spine pain, rheumatism, crooked knee	10
Respiratory tract diseases	Sore throat, flu, cough, throat inflammation, asthma, asthma crisis	7
Undefined diseases	Chest lump, inflammation, cracked heels, itching, healing	5
External causes of morbidity and mortality	Swelling, wound, barbs, thorn removal	4
Injury, poisoning, and some other consequences of external causes	Bruise, arm dislocation, ankle fracture	3
Diseases of the circulatory system	Hemorrhoids, stroke, thrombosis	3
Skin and subcutaneous tissue disorders	Boil, blackhead, acne	3
Nervous system disorders	Headache	1
Ear and mastoid apophysis disorders	Ear pain	1
Digestive tract diseases	Constipation	1

### Relative importance

In order to assess which species are of greatest cultural importance to informants, the relative importance (RI) of the species (adapted from Bennett and Prance [45]) was calculated. The value is obtained by the formula: RI = NBS + NP, where NBS = number of body systems (disease categories), which is given by the number of body systems treated by a particular species (NBSS) over the total number of body systems treated by the most versatile species (NBS = NBSS/NBSVS), and NP = number of properties, which is given by the number of properties assigned to a given species (NPS) over the total number of properties assigned to the most versatile species (NPVS) NP = NPS/NPVS.

## Results and discussion

The use of 22 animals as a medicinal resource was recorded from the informants (Table 2). Of this total, 19 are vertebrates and three invertebrates, distributed in eight taxonomic categories, of which mammals (*n* = 6 species), reptiles (*n* = 6), and fish (*n* = 4) were the most important. It is not surprising, given that vertebrates are the most commonly used animals in Brazilian folk medicine [29]. The prominence of the recorded taxonomic categories in the surveyed area has also been recorded in several studies, demonstrating their relevance as a therapeutic resource [23, 33, 36, 46–53].

**Table 2:** Zootherapies used in the fishing community of Diogo Lopes, Macau - RN

Class/family/species/“local name,” popular name (En/US)	Number of citations	Parts used	Diseases	Disease categories	IUCN (2018)	CITES	Brazilian list MMA (2014)
Mammalia							
Bovidae							
*Ovis aries* Linnaeus, 1758—“carneiro,” sheep	24	Fat, suet	Bruise, arm dislocation, ankle fracture, leg pain, swelling, plantar fasciitis, “hard nerves,” joint pain, knee pain, bone pain, hardened joints, crooked knee, cracked heels	Injury, poisoning and some other consequences of external causes, musculoskeletal and connective tissue disorders, undefined diseases	0	0	0
Canidae							
*Canis familiaris* Linnaeus, 1758—“cachorro,” dog	1	Feces	Throat inflammation	Respiratory tract diseases	0	0	0
*Cerdocyon thous* (Linnaeus, 1766)—“raposa,” crab-eating fox	15	Fat, leather	Sore throat, throat inflammation, hemorrhoids, inflammation	Respiratory tract diseases, diseases of the circulatory system, undefined diseases	LC	II	0
Cervidae							
*Mazama* sp.—“veado,” deer	1	Fat	Leg pain	Musculoskeletal and connective tissue disorders	0	0	0
Dasypodidae							
*Euphractus sexcinctus* (Linnaeus, 1758)—“tatu-peba,” six-banded armadillo	2	Fat	Thorn removal, ear pain	External causes of morbidity and mortality, ear and mastoid apophysis disorders	LC	0	0
Delphinidae							
*Sotalia guianensis* (P.-J. van Bénéden, 1864)—“boto-cinza,” Guiana dolphin	4	Fat	Itching, throat inflammation, leg pain, “hard nerves”	Undefined diseases, respiratory tract diseases, musculoskeletal and connective tissue disorders	DD	0	VU
Birds							
Cathartidae							
*Coragyps atratus* (Bechstein, 1793)—“urubu-de-cabeça-preta,” American black vulture	1	Liver	Asthma	Respiratory tract diseases	LC	0	0
Phasianidae							
*Gallus gallus* (Linnaeus, 1758)—“galo,” chicken	11	Fat	Sore throat, throat inflammation, boil, chest lump, inflammation, constipation	Respiratory tract diseases, skin and subcutaneous tissue disorders, undefined diseases, digestive tract diseases	LC	0	0
Reptilia							
Boidae							
*Boa constrictor* Linnaeus, 1758—“jiboia,” boa	2	Fat, oil	Ear pain, spine pain	Ear and mastoid apophysis disorders, musculoskeletal and connective tissue disorders	0	0	0
Cheloniidae							
*Chelonia mydas* (Linnaeus, 1758)—“tartaruga-verde,” green turtle	17	Fat, oil	Throat inflammation, bone pain, spine pain, rheumatism, stroke, thrombosis, boil, healing	Respiratory tract diseases, musculoskeletal and connective tissue disorders, diseases of the circulatory system, skin and subcutaneous tissue disorders, undefined diseases	EN	0	VU
Iguanidae							
*Iguana iguana* (Linnaeus, 1758)—“Camaleão,” common green iguana	9	Fat, bones	Sore throat, throat inflammation, barbs, hemorrhoids	Respiratory tract diseases, external causes of morbidity and mortality, diseases of the circulatory system	0	0	0
Teiidae							
*Salvator merianae* (Duméril & Bibron, 1839)—“lagarto teju,” tegu lizard	55	Fat	Sore throat, throat inflammation, wound, ear pain, knee pain	Respiratory tract diseases, external causes of morbidity and mortality, ear and mastoid apophysis disorders, musculoskeletal and connective tissue disorders	LC	0	0
Tropiduridae							
*Tropidurus hispidus* (Spix, 1825)—“lagartixa de lajedo,” Peters’ lava lizard	4	Meat, blood, head	Sore throat, throat inflammation	Respiratory tract diseases	0	0	0
Viperidae							
*Crotalus durissus* Linnaeus, 1758—“cascavel,” South American rattlesnake	6	Fat, oil	Hardened joints, bone pain, spine pain, blackhead, acne, cracked heels, headache	Musculoskeletal and connective tissue disorders, skin and subcutaneous tissue disorders, nervous system disorders	LC	III	0
Amphibia							
Bufonidae							
*Rhinella jimi* (Stevaux, 2002)—“sapo cururu,” frog	1	Fat	Throat inflammation	Respiratory tract diseases	LC	0	0
Elasmobranchii							
Carcharhinidae							
*Rhizoprionodon lalandii* (Müller & Henle, 1839)—“tubarão,” Brazilian sharpnose shark	2	Liver oil	Stroke, healing	Diseases of the circulatory system, undefined diseases	DD	0	0
Actinopterygii							
Echeneidae							
*Echeneis naucrates* Linnaeus, 1758—“rêmora,” whitefin sharksucker	1	Sucker/fixer part	Asthma crisis	Respiratory tract diseases	LC	0	0
Scombridae							
*Scomberomorus cavalla* (Cuvier, 1829)—“cavala,” king mackerel	1	Posts	Asthma crisis	Respiratory tract diseases	LC	0	0
Syngnathidae							
*Hippocampus reidi* Ginsburg, 1933—“cavalo-marinho,” Long-snout Seahorse	1	Whole body	Asthma crisis	Respiratory tract diseases	NT	0	VU
Insecta							
Apidae							
Bee	7	Honey, gel	Flu, cough, leg pain	Respiratory tract diseases, musculoskeletal and connective tissue disorders	0	0	0
Malacostraca							
Ocypodidae							
*Ocypode quadrata* (Fabricius, 1787)—“caranguejo maria-farinha,” ghost crab	1	Whole body	Asthma crisis	Respiratory tract diseases	0	0	0
Hydrozoa							
Physaliidae							
*Physalia physalis* (Linnaeus, 1758)—“caravela-portuguesa,” Portuguese man-of-war	1	Whole body	Wounds	External causes of morbidity and mortality	0	0	0

The most cited species by the individuals interviewed were *Salvator merianae* (Duméril and Bibron, 1839)—teju (55 citations), *Ovis aries* (Linnaeus, 1758)—sheep (24 citations), *Chelonia mydas* (Linnaeus, 1758)—green turtle (17 citations), *Cerdocyon thous* (Linnaeus, 1758)—fox (15 citations), and *Gallus gallus* (Linnaeus, 1758)—chicken (11 citations). The significant number of citations of *S. merianae* confirms the importance of the species as a therapeutic resource in the study area. Its medicinal use has been registered in several localities of the country [54–59]. The data show a prevalence of wild species (*n* = 19) being used as therapeutic resources when compared with domestic species (*n* = 3), corroborating the results reported by Alves and Rosa [33] on the predominant use of wild species in the folk Brazilian medicine. This trend has been recorded in various medical systems around the world [24, 30, 60–63].

Regarding the relative importance of the species, although *C. mydas* (green turtle) was not the most cited by informants, it was the most used for a wide range of diseases, presenting an RI = 1.6. It was considered the most versatile species for multiple disease prescriptions, followed by sheep (*O. aries*), RI = 1.5; chicken (*G. gallus*), IR = 1.1; teju (*S. merianae*), RI = 1.0; and rattlesnake (*Cortiles durissus* Linnaeus, 1758), RI = 1.0 (Table 3).

**Table 3:** Relative importance of the most versatile medicinal animal species in the fishing community of Diogo Lopes - RN, Brazil

Species	Habitat	Relative importance
*Chelonia mydas*	Aquatic	1.6
*Ovis aries*	Terrestrial	1.5
*Gallus gallus*	Terrestrial	1.1
*Salvator merianae*	Terrestrial	1.0
*Crotalus durissus*	Terrestrial	1.0
*Iguana iguana*	Terrestrial	0.8
*Cerdocyon thous*	Terrestrial	0.8
*Sotalia guianensis*	Aquatic	0.8
Bee (Apidae)	Terrestrial	0.5
*Boa constrictor*	Terrestrial	0.4
*Euphractus sexcinctus*	Terrestrial	0.4
*Tropidurus hispidus*	Terrestrial	0.3
*Echeneis naucrates*	Aquatic	0.2
*Ocypode quadrata*	Aquatic	0.2
*Hippocampus reidi*	Aquatic	0.2
*Physalia physalis*	Aquatic	0.2
*Rhinella jimi*	Terrestrial	0.2
*Rhizoprionodon lalandii*	Aquatic	0.2
*Mazama* sp.	Terrestrial	0.2
*Coragyps atratus*	Terrestrial	0.2
*Scomberomorus cavalla*	Aquatic	0.2
*Canis familiaris*	Terrestrial	0.2

Among the animals listed in this study, 14 are terrestrial, most of them from the dry forest environment. Additionally, the use of aquatic animal species from marine/estuarine habitats was also reported (*n* = 8). Among these, the species with the highest importance index (RI) was the green turtle *C. mydas*. The data suggest that human communities tend to use medicinal animals in accessible environments in local ecosystems. As pointed out by Alves and Rosa [23], the use of local resources that are more easily accessible is probably related to cultural and historical aspects. This is because medicinal knowledge is focused on species that locals are familiar with, reflecting the transmission of knowledge across generations as well as financial constraints that limit access and use of other resources. This relationship between medicinal use and local knowledge has been recorded in several studies in different parts of the world. Adeola [64] noted that in Nigeria, the use of wild animals is linked to the environment in which people live and the relative abundance of species in that environment. Similar situation was recorded in India [24, 65] and Argentina [14, 25]. In Brazil, studies carried out in fishing communities have documented the strong tendency of aquatic animals to be used in folk medicinal practices [23, 32, 33, 52, 66, 67]. On the other hand, studies developed in populations of semiarid regions indicate the prevalence of terrestrial or endemic animals from these regions [30, 34–36, 59].

From the total number of records, it was possible to identify 14 products from whole animals or parts of their bodies, which are used to treat 38 diseases diagnosed by the community (Table 2). As for the methods of preparation of these products, the following were recorded: whole animals or parts are generally roasted and macerated and the resulting powder is ingested in the form of teas. Animal metabolism secretions such as lard, gel, blood, and tallow are used as ointments to massage the affected area or ingested pure or with coffee. Among these zootherapeutic products cited by informants, lard stood out as one of the most commonly used products (number of citations = 120), especially teju’s lard (*S. merianae*) which was reported to be widely used to treat throat problems. Most informants (78%) who cited the use of lard reported that it needs to be melted or heated and taken pure in the form of oil. When used externally, the lard is applied to the wound. According to Alves et al. [68], the frequency in which lard is used can be attributed to the fact that the main animals used are vertebrates, which have a large amount of fat in their body. Previous work has also reported the use of lard as the most commonly used raw material in the treatment and cure of various diseases [47, 50, 69, 70].

The categories of diseases with the highest number of citations were respiratory tract diseases (92 citations) and musculoskeletal system and connective tissue diseases (35 citations). The diseases with the highest number of citations were throat inflammation (54 citations) and sore throat (27 citations). This trend registered in the present study corroborates the pattern pointed out in other cities of the Northeast region, indicating that these categories are widely treated with medicinal animals [33, 58, 68, 71]. Additionally, according to the informants, it was possible to register the use of the same species in the treatment of more than one disease. An example was the ram (*O. aries*), whose parts (tallow and lard) are used to treat various illnesses such as bruise, arm twists, ankle fracture, leg pain, swelling, plantar fasciitis, joint pain, knee pain, cracking heels, bone pain, hard joints, and bent knee. Another animal of multiple medicinal uses in the study area is the turtle (*C. mydas)* which is used to treat throat inflammation, bone pain, stroke, back pain, rheumatism, boil, and thrombosis, being also used for healing. Other species have also been reported for various therapeutic indications: teju (*S. merianae*), chicken (*G. gallus*), chameleon (*Iguana iguana* Linnaeus, 1758), fox (*C. thous*), and rattlesnake (*C. durissus*).

The use of zootherapeutic products may be related to the use of resources that would otherwise be wasted [69]. According to these authors, populations tend to use leftovers of food for therapeutic purposes. Not surprisingly, therefore, several of the medicinal animals are hunted or fished by the local population for food purposes. An example is the teju (*S. merianae*), which represents an important source of protein and is one of the most hunted species used as food in traditional and/or indigenous communities [72], and its leftovers such as lard, tail, and tongue are used as medicines.

According to most informants, the use of animals listed as a medicinal resource was a common practice in the past and was most often obtained through hunting or given by older people (parents, grandparents, great-grandparents, or hunter friends). When asked about the preference for treating a disease, they reported that they preferred the folk medicine (from animals or plants) to medicines sold in pharmacies, but it was currently very difficult to get the animal. As pointed out by Alves et al. [54], some factors contribute to the reduction of use of zootherapics and herbal medicines. Among them are the decline of fauna and flora due to deforestation, burning, and hunting, and the presence of health centers with free distribution of medicines. This reduction in the use of zootherapics was also reported by informants in the study by Lima and Santos [73], who recorded that the species were decreasing as a consequence of hunting and deforestation activities for development.

Among the medicinal species recorded in the present study, the seahorse (*Hippocampus reidi* Ginsburg, 1933), the turtle (*C. mydas*), and the dolphin (*Sotalia guianensis* (P.-J. van Bénéden, 1864)) are present in the Red List of Threatened Species of the International Union for Conservation of Nature (IUCN) [43] and the List of Endangered Brazilian Fauna Species [44]. The impacts of zootherapeutic practices on wild populations should be carefully investigated, since, unlike herbal remedies, the use of zootherapeutic products most often occurs after an animal is sacrificed [14]. However, it is important to point out that, despite being used in folk medicine, the impacts of zootherapeutic practice on threatened species are not significant, especially when compared with other factors triggering population decline such as habitat degradation and capture of these animals for other purposes that are not necessarily medicinal [74]. According to Alves et al. [75], understanding the trend and multiplicity of therapeutic use of animals is a particular concern from a conservationist point of view. This is noteworthy because threatened species such as those reported in this and other studies can be replaced by non-threatened species with similar properties.

Given the information obtained from the informants, it is noticeable that there is a tendency of using medicinal animals that occur near the sampled locality. This was especially clear in relation to species that are targeted for hunting and fishing, showing that the environment directly influences the choice of zootherapeutic resources and the medicinal use represents a strategy of optimizing the use of resources. Zootherapeutic practices are related to ecological, cultural, historical, sociological, economic, and health aspects [58, 76], connecting people to the environment and enriching local knowledge [77, 78].

## Data Availability

All data generated or analyzed during this research are included in this published article.
